# No significant change in domestication-admixture during the marine phase of an Atlantic salmon population

**DOI:** 10.1038/s42003-026-10051-z

**Published:** 2026-04-16

**Authors:** Marine S. O. Brieuc, Francois Besnier, Alison C. Harvey, Per Tommy Fjeldheim, Kaja C. Andersen, Vidar Wennevik, Kjell R. Utne, Monica F. Solberg, Fernando Ayllon, Sofie Knutar, Anne Grete Sørvik, Laila Unneland, Øystein Skaala, Kevin A. Glover

**Affiliations:** Institute of Marine Research, Bergen, Norway

**Keywords:** Conservation biology, Ecological genetics

## Abstract

Domesticated organisms have been pivotal in food-production and human evolution. Yet, interbreeding between domesticated and wild conspecifics may have significant negative impacts on native populations. Atlantic salmon, subjected to more than 50 years of domestication and extensive escapes from aquaculture, represents a flagship example of this type of interaction. However, currently no data documents the influence of introgression on survival of a naturally-recruiting wild population during the marine phase of the life-cycle. Following analysis of 885 out-migrating smolts and 7275 adults returning to a Norwegian river in the period 2016–2023, we find no statistical evidence that marine survival varies with domestication-admixture, as admixture levels are not significantly different between out-migrating smolts and returning adults. As this wild population is highly admixed (25–30%) following extensive intrusion of escapees during 1989–2012, we conclude that in the current evolutionary state of this population, i.e. several generations after introgression started, there is no statistical evidence of strong selection against admixed fish during the marine phase. This is likely to be driven by natural selection purging mal-adapted individuals during the freshwater stage where fitness differences are well-documented, and potentially, also in the marine stage during the initial generation(s) following introgression.

## Abstract (web summary)

Long-term monitoring of an Atlantic salmon population reveals no effect of domestication-admixture on marine survival, suggesting natural selection may mitigate the long-term fitness costs of introgression in the marine phase.

## Introduction

As the world´s living resources come under unprecedented pressure from the expanding human population, global food production is becoming increasingly dependent on the use of domesticated plants and animals^1–3^. However, while domestication represents an important pillar in the development of the modern world, this has not come without challenges.

In addition to expanding land area for feed growing at the expense of natural habitats and overfishing of ecologically important species to produce aquaculture feed^4,5^, one of the main environmental consequences of rearing domesticated organisms is their escape into the natural environment, and thereafter interbreeding with wild conspecifics, as has been observed across multiple taxa^6–9^. This situation is prominent in fishes, and especially in Atlantic salmon (*Salmo salar*) where nearly half a century of escapes from cage-based aquaculture installations^10–12^ has left a legacy of introgression in native populations throughout their native range^13–15^. Due to the extent of this interaction, and that the Atlantic salmon is one of the most domesticated fish^2^, the situation observed in this species may be regarded as the flagship example of this type of interaction^9^.

Understanding the consequences of domestication-introgression in native populations is of interest to evolutionary biologists and natural resource managers alike. Extensive research has demonstrated that the offspring of domesticated Atlantic salmon display lower relative survival in comparison with wild salmon in rivers^16–19^. Lower relative survival of domesticated offspring has also been reported beyond the F1 generation^20^, and also for admixed individuals identified in wild populations^21,22^. However, estimates of relative survival during the marine phase of the life cycle are both more sparse and inconsistent. Out of two common garden studies performed in the natural environment, one observed lower marine survival of the offspring of domesticated and hybrid fish^19^, while another did not^17^. Release studies using hatchery-produced smolts of domesticated, domesticated x wild and wild parents have shown lower survival of fish of domesticated background in the marine phase^19,20,23^. However, those fish had not undergone natural selection during the freshwater phase, and as such, may not correctly reflect the situation following introgression in the wild. Furthermore, releases have identified maternal and/or non-additive inheritance patterns^19,23^, complicating matters.

**Fig. 1:**
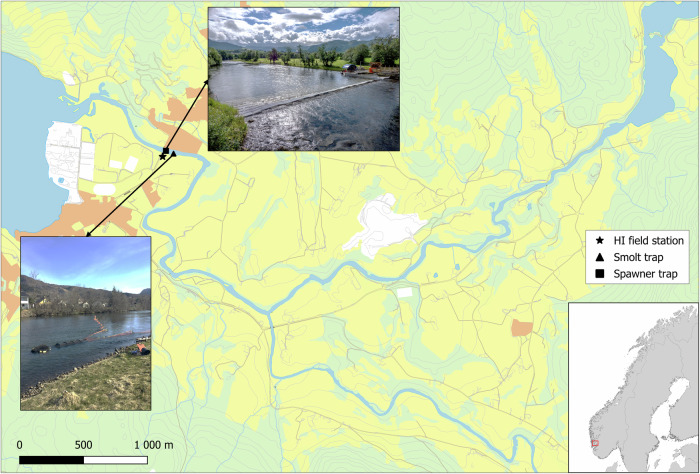
Location of the river Etne and sampling locations used in the current study. The location of the smolt traps and spawner traps are indicated on the map, along with pictures of the traps.

Marine survival, a key component of Atlantic salmon productivity, has been decreasing across the species distribution in the last few decades^24,25^. However, the influence of domestication-introgression on survival during the marine phase in natural populations is still unclarified and in need of further comprehensive investigation. Here we aimed to characterize the effect of admixture on marine survival in a highly domestication-admixed wild salmon population. The river Etne (Fig. 1), Norway, is home to an Atlantic salmon population that has been extensively subjected to domesticated escapees in the period 1989–2012^11^, stopped only in the period 2013-present due to the installation of an upstream fish trap and individual sorting of all salmon entering the river that effectively removes nearly all farmed escapees entering the river^26,27^. To characterize the contemporary effects of introgression on marine survival, several generations after introgression started, we directly compared individual-fish admixture between 885 PIT tagged out-migrating smolts in the period 2016–2020, and those returning as PIT tagged adults (30). We also compared admixture between the PIT-tagged smolts migrating out of the river in the period 2016–2020 (885 fish) against all adults (PIT- and non-tagged) returning to spawn in the river in 2017–2023 that were aged and thus assigned to the five outward migrating smolt cohorts 2016–2020 (7275 adults).

## Results

### Direct observations of returning adults tagged as smolts

For the smolt cohorts migrating out of the river in 2016–2020, total marine survival, based upon recapture of PIT-tagged fish, ranged from 1.1 to 5.9% per smolt year, independent of the number of years spent at sea. Corrected mean admixture across all years in smolts migrating from the river was 28.2%, while that of the survivors was 25.7% (Table 1). After 10,000 bootstraps without replacement, the observed mean admixture of the survivors was within the range expected under the hypothesis of no difference in admixture between all out-migrating smolts and the survivors of the marine phase. This was the case for all the years combined and for all individual years except 2018 (where it was lower than expected, but the number of survivors that year was only 4, so this result should be interpreted with caution). Thus, these direct observations do not indicate statistically significant selection against admixture in this phase of the life cycle (Table 1).

**Table 1:** Marine survival: number of individuals sampled as smolts and observed as adults; sample sizes correspond to individuals with less than 50% MV with the microsatellites (sample size with the 130 SNP panel in parenthesis), observed and expected range of mean admixture based on the number of observed fish returning each year (10,000 bootstraps). Standard deviation for the observed mean is indicated in parentheses

Smolt Cohort	Number of smolts sampled	Number of returning individuals	Marine survival	Observed mean of admixture of smolts (SD)	Observed mean of admixture of returning adults (SD)	Expected range of admixture (5th–95th percentiles)
2016	185 (143)	9 (6)	4.9%	0.3431 (0.1440)	0.3828 (0.0804)	0.2316–0.4586
2017	187 (183)	8 (8)	4.3%	0.3277 (0.1461)	0.3367 (0.1310)	0.2312–0.4301
2018	183 (185)	5 (4)	2.7%	0.3109 (0.1561)	0.1448 (0.1524)	0.1644–0.4648
2019	187 (186)	11 (10)	5.9%	0.3015 (0.1503)	0.2633 (0.1526)	0.2132–0.3918
2020	185 (188)	2 (2)	1.1%	0.3374 (0.1367)	0.4789 (0.0190)	0.1534–0.5175
All years	927 (885)	35 (30)	3.8%	0.3232 (0.1474)	0.3053 (0.1505)	0.1455–0.5140

**Fig. 2:**
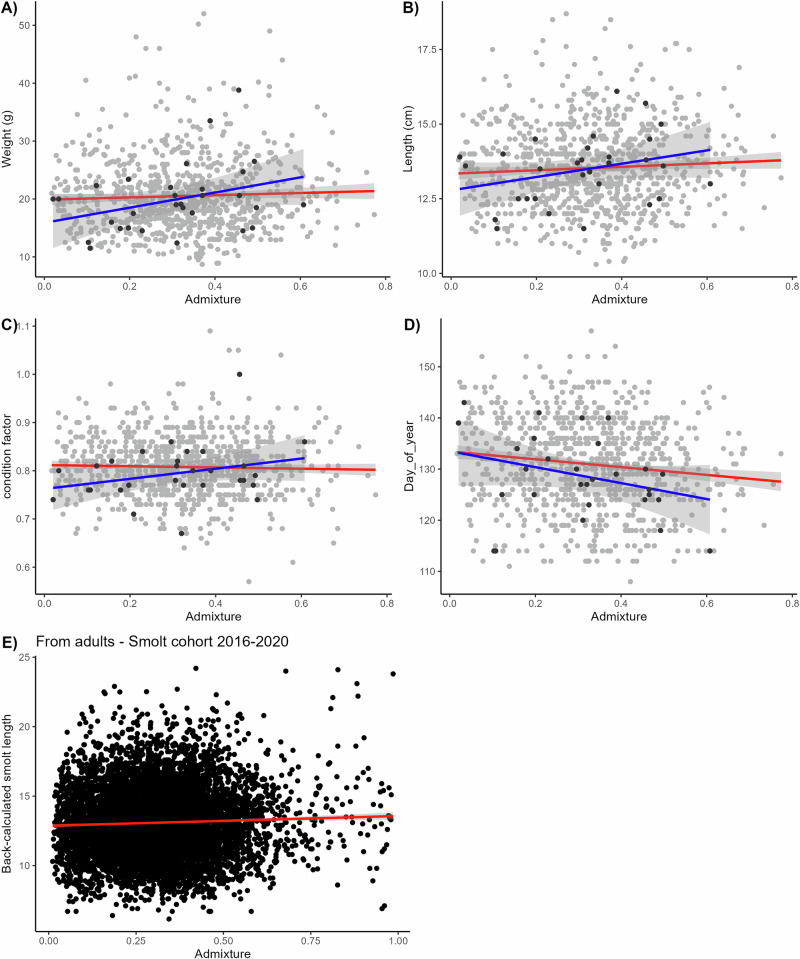
Linear regressions of length(cm), weight (g), condition factor and outmigrating day against admixture rate. **A**–**D** Regressions for the individuals that were sampled as smolts (gray, *n* = 885) and those that returned as adults (black, *n* = 30); the red line is the linear regression of all the smolts sub-sampled and genotyped while the blue line corresponds to the linear regression of the survivors only. All but one of the regressions were significant : *p* value for weight: 0.103 (all) and 0.073 (returned), *p* value for length: 0.090 (all) and 0.113 (returned); *p* value for condition factor: 0.540 (all) and 0.139 (returned); *p* value for outmigrating day: 6.57e-05 (all) and 0.125 (returned). **E** Linear regression of back-calculated smolt lengths against admixture for all the adults from smolt cohorts 2016–2020 sampled in Etne. *p*-value: 0.000236 (*n* = 7275).

No significant correlation was found between individual smolt weight, length or condition factor and admixture for all the smolts (885 fish), nor for the smolts that survived to return as adults (30 individuals) (Fig. 2a–c). However, there was a significant negative correlation between admixture and out-migrating date for all the smolts, but not for the smolts that survived as adults (Fig. 2d). In contrast, when using the larger dataset consisting of back calculated length at smolt age for all the returning adults assigned to smolt cohorts 2016–2020 (more than 7000 individuals), we observed a significant positive effect of admixture on smolt length (*p* < 0.001, slope = 0.70086, Fig. 2e). When randomly resampling the dataset with 885 individuals without replacement, we determined that the absence of a significant relationship when using the smolts was attributed to the difference in sample size given the high variability in smolt size and the small size effect of admixture on smolt size (Fig. S1).

### Changes in admixture between smolts and adults

**Fig. 3:**
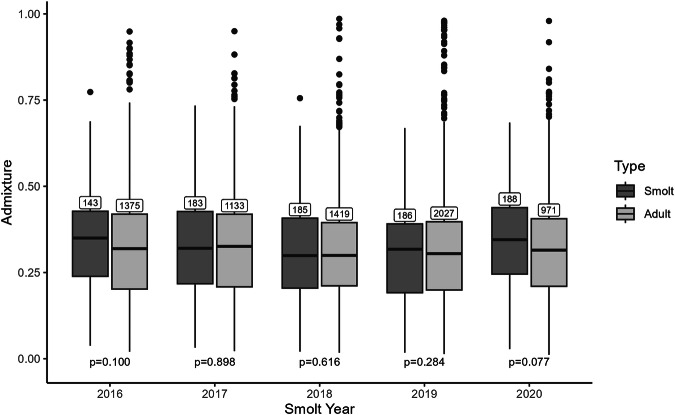
Distribution of admixture for smolts and adults by Smolt Cohort. *p* values for the two-sided Kolmogorov-Smirnov test for each smolt cohort is indicated below the boxplots. Sample sizes are indicated in boxes above each boxplot. Adults with admixture close to one could be the offspring of two domesticated fish that successfully mated before the trap was installed, or be the occasional farmed fish that may have bypassed the trap in case of flooding event for example.

From scale readings, the sea age was determined with high confidence (Table S1) for over 98% of the adults returning to the river Etne from 2017 to 2023. This resulted in a sample size of 7275 adults that were assigned to smolt cohorts 2016–2020. Yearly adult sample sizes ranged from 986 (smolt cohort 2020) to 2116 (smolt cohort 2019). The corrected mean admixture for the smolt cohorts ranged from 25.1 to 31%, and those of the adults ranged from 25.8 to 28.2% (Fig. 3). For all five smolt cohorts investigated, there was no significant difference in admixture distributions computed for the out-migrating smolts and the corresponding returning adults (*p*-values for the two-sided Kolmogorov-Smirnov Test: 0.077–0.898; Fig. 3).

When all smolts and adults were separated by genetic sex (Fig S2), we did not observe significant differences in distribution of admixture for any of the smolt cohorts for either sex (two-sided Kolmogorov-Smirnov Test nominal *p* = 0.049 for the females in 2016, but not significant after correction for multiple testing across 5 years). Similarly, there were no significant differences in level of admixture with sea-age for the returning adults compared to the smolt cohorts (Fig S3). However, when examining the probability of returning as 1SW or MSW, we observed a significant effect of admixture on the probability of early maturation in males only (significant interaction between admixture structure and male sex: (Estimate = 1.38480, *p* = 0.00472) (Tables S2, S3)

## Discussion

The present study is, to the best of our knowledge, the first to thoroughly investigate the effect of domestication-admixture on the survival of naturally recruited Atlantic salmon smolts in the marine environment. Using a dataset consisting of 885 smolts migrating out of the river Etne in the period 2016–2020, and 7275 age-determined adults returning to the river in the years 2017–2023, we did not detect statistically significant changes in admixture in the population during the marine phase, indicating that individual-fish admixture does not have a strong effect on marine survival in this population’s current evolutionary state. The results of this unique study, made only possible by the highly extensive and long-term sampling in the river Etne via the trapping facility, have significant implications for how we view the long-term influences of domestication-admixture on wild salmon populations.

In general, larger smolts, and fish returning after just 1 year in the sea, exhibit higher marine survival rates compared to smaller smolts, and fish returning after multiple sea winters^28,29^. Several studies have reported that domestication-admixture may result in both younger and larger smolts^18,19,30^. Indeed, we also observed slightly larger smolt sizes for admixed fish using back-calculated smolt lengths from adults and a positive effect of admixture on probability of early maturation in males. Theoretically, these observations could have led to increased marine survival of highly admixed fish. However, we did not observe this in the current study. This highlights that while larger smolts and early maturing fish may have higher survival for wild fish, this may not be the case if increased smolt size and early maturity are the results of domestic influence. Potentially, any such advantage of increased smolt size and/or earlier maturity may have been countered by the other aspects of the more heavily domestication-admixed fish, such as a genetically lower propensity to survive that has at least been documented in the freshwater phase of the life cycle.

Reduced survival of domesticated and domestication-admixed Atlantic salmon has been consistently demonstrated in the freshwater stage of the life cycle, under different scenarios such as first-generation hybrids, single large scale introgression events, or moderate levels of introgression over large time scales^17–22,31^. Here, and in contrast to what has been observed in the freshwater stage, we do not detect statistical evidence of reduced survival of domestication-admixed salmon during the marine phase in the current evolutionary state of the studied population. This result is consistent with the findings of Fleming et al.^17^, where 22 farmed and 17 wild individuals were transplanted into the river Imsa in Norway and the composition (wild, farmed, hybrids) of the population was followed during the different life stages up to adulthood. There, selection against hybrids and domesticated individuals was detected in the early freshwater stage, but not during the marine phase. Other studies, based upon release of hatchery-reared smolts and/or release of eggs followed through the entire life-cycle, have shown reduced survival of offspring of domesticated parents during the marine phase^16,19,20^. However, data from smolt-releases are not directly transferable to real life scenarios as the fish have not undergone strong natural selection, typically at 95–98% mortality^18,19^, during the freshwater phase. In addition, substantial non-additive variation has been observed for survival during the marine phase of the life cycle^19,23^. In particular, one of the studies that included both naturally recruited smolts (planted as eggs) and hatchery-produced smolts, observed consistently higher marine survival of F1 hybrids than offspring from either wild or domesticated parents^19^. Clearly, and in contrast to the extensive and consistent results from the freshwater phase of the life cycle, documentation of the influence of admixture on marine survival during the first generation of introgression is variable, potentially caused by different study designs. We therefore propose that after one or more generations of natural selection following introgression, throughout both the freshwater and possibly marine phases of the life cycle, any fitness costs experienced during the initial marine phase are likely to be alleviated in the long term. This is consistent with the long-term predictions from the IBSEM model that indicate limited productivity losses in wild populations following introgression of domesticated escapees unless at consistently high intrusion levels^32,33^. While this remains to be investigated in other systems, the trapping facility and full-population sampling used in the river Etne provide a quality and volume of data that exceeds what is possible to gain from introgressed rivers that lack such infrastructure. Nevertheless, we were unable to reject the null hypothesis of no selection against domestication-admixture. Therefore, our data do not rule out the possibility of a low level of selection that the study lacked the power to detect.

The wild Atlantic salmon population inhabiting the river Etne has been subjected to extensive spawning intrusion from farmed escapees in the period 1989–2012^11^ which has cumulated in a high (~25–30%) level of introgression^30^. This gives the river status as one of the most highly domestication-introgressed populations in Norway^15^, and most likely globally. Based upon our observations, we conclude that the extensive input of genetic material from farmed escapees during that 20+ year period has been countered by the process of natural selection purging mal-adapted individuals, leaving the population in its current state, i.e., without any detectable influence of admixture on marine survival. Selection would have happened during the freshwater and possibly marine phase of the life cycle, and constantly throughout that 20+ year period, reflecting a dynamic introgession-selection “tug-of-war”. If the observations in the river Etne reflect a typical response of Atlantic salmon populations to similar scenarios, our results suggest that the extensive level of gene-flow observed in many wild populations in Norway^15,34,35^, and potentially in other countries where this occurs^31,36,37^, may not have a strong long term effect on the marine survival of these populations, at least in the absence of continued introgression. That is, so long as the population is robust enough to survive any potential short-term fitness and productivity consequences of introgression^17,18,20^ while natural selection removes the most maladapted individuals from the population.

## Methods

### The river Etne and sampling

We have complied with all relevant ethical regulations for animal use. The research permit for this study was delivered by the Norwegian Food Safety Authority and is valid for all activities at the field station. All the samples in this study were collected in the river Etne, located in western Norway (Fig. 1). This river was subjected to high levels of introgression from domesticated individuals since 1989^11^, and has been estimated to be ~25% introgressed^30^. An upstream fish trap was installed in the lower reaches of the river in 2013 to prevent escaped domesticated salmon from entering the river^26^. This trap permits sampling of the entire wild adult population and effectively blocks further introgression from domesticated salmon in the population. Once sampled in the trap, phenotypically wild salmon are released above the trap to continue their spawning migration while domesticated escapees (identified by outward characteristics such as pigmentation patterns and body shape that differ between wild and domesticated individuals) are removed from the river. Scales for all farmed and wild fish are later used to validate types. Sampling in the trap consists of extensive phenotypic measurements in addition to scale samples for obtaining life-history information and a tissue sample for DNA analysis (Fig. 1). Adults were not anesthetized as sampling was done quickly (less than 2 min per fish) and directly in the river, and anesthesia was considered more disturbing to the fish in this scenario.

In the years 2016–2020, 1300–2800 smolts migrating from the river were randomly sampled in a smolt trap per year, corresponding to 3–5% of all the smolts (Table S4). Smolts were anesthetized by water batch with benzocaine (30–40 mg/L), PIT tagged, measured (weight and length), and tissue sampled (fin clipped) before being released below the smolt trap to continue their migration to the ocean. Of these, around 180 smolts per year (~900 in total), corresponding to an estimated 0.2–0.5% of all smolts (Table S4) were genotyped and thereafter used for detailed analysis in this study.

After having spent 1–3 years in the sea, adults from the smolt cohorts in 2016–2020 returned to the river in the years 2017–2023. This totaled 12,831 adults and represents almost the entire adult population of this river (as a small proportion of the individuals may bypass the trap during flooding events). As these fish were aged by scale reading (freshwater age and sea age), it was possible to identify their smolt year. Individuals that were assigned to the smolt cohorts 2016–2020 (7275 fish) were subsequently used to compare admixture profiles of fish migrating out of the river, and back into the river, to estimate marine mortality and its association with estimates of individual-fish admixture.

### Genetic analysis and estimation of admixture

For all individuals in this study, DNA was isolated using DNAdvance Kit (Beckman Coulter, Inc.). All samples were genotyped at 31 microsatellite loci and the sex determining gene *sdY*^26,38,39^ according to protocols described in supplementary Tables S5–S6. PCR products were analyzed with ABI 3720 Genetic Analyzer and genotypes were called using GeneMapper version 6 software (Applied Biosystems). Individuals that had more than 50% missing genotypes were removed from the dataset. In addition, all individuals were genotyped with a panel of 130 SNPs to permit the estimation of individual-fish domestication-admixture (i.e., proportion of their genome derived from domesticated fish). This panel of SNPs, that are collectively diagnostic for farmed vs wild salmon in this population, was mined from a pool of 50,000 SNPs presented in the Besnier et al.^30^ study on this population (Figs S4, S5 and Supplementary Material). Genotyping was conducted on a Sequenom MassARRAY analyzer (San Diego, CA, USA) and the list of primers and extensions primers used are described in Supplementary Data 1. Admixture for all smolts and adults sampled in this study was estimated with the program Structure^40^, using 100,000 burnin and 500,000 MCMC iterations for all individuals with less than 50% missing genotypes. The genetic assignment was performed for each test individual separately by comparing the test individual’s genotype with a panel of wild and domesticated reference individuals described in Besnier et al.^30^. This was achieved by using Structure with population information (Structure parameter “popinfo = 1” and “popflag = 1”) to provide a prior genetic assignment for the reference individuals and no prior for the test individual. For consistency between studies, population average admixture values were corrected according to Karlsson et al.^41^ to account for the fact that average admixture of reference wild fish was larger than zero and average admixture for domesticated individuals was lower than one.

### Effect of admixture on marine survival

We used two complementary approaches to test the effect of admixture on marine survival. First, admixture was compared between individuals sampled as PIT-tagged smolts on their way out of the river in the period 2016–2020 (885fish) and those returning as PIT tagged adults in 2017–2023 (30 individuals). Second, by comparing admixture between the PIT-tagged smolts migrating out of the river in the period 2016–2020 (885 fish) against all adults (PIT- and non-tagged) returning to spawn in the river in 2017–2023 that were aged and thus assigned to the five smolt cohorts 2016–2020 (7275 adults).

#### Direct observations of returning adults tagged as smolts

Our first aim was to determine, from the individuals sampled as smolts, if survivors returning as adults displayed higher or lower admixture than the pool of smolts from which they were sampled. Individuals surviving the marine phase were directly identified by their PIT-tag during adult sampling. However, as PIT-tags can be lost e.g., ref. ^42^ we also used genetic analysis to identify smolts surviving to return as adults. To do so, we used the microsatellite data for all fish sampled in this study, and identified direct matches in the parentage and sibship program Colony v.2.0.7.1^43^. The individuals that were sampled both as smolts and returning adults were identified using the option “Clone inference”.

Among the 183–187 smolts sampled annually in the period 2016–2020, 2–11 individuals from each cohort (identified by PIT tags and genetics) were observed as adults entering the river in subsequent years (Table 1). From there marine survival was estimated as: $\frac{{number\; of\; individuals\; sampled\; as\; smolt}}{{number\; of\; individuals\; sampled\; as\; smolt\; and\; observed\; again\; as\; adult}}$. We did not take into account harvest in our calculations, as there is no sea fishery, and while there is some recreational fishery in the river, it is limited and most of it is occurring above the trap, where fish would have already been sampled, therefore not affecting the marine survival estimates. To assess whether the admixture observed among individuals surviving the marine phase significantly differed from random expectations in either direction, we employed a two-tailed permutation test with 10,000 iterations for each smolt cohort, as well as for all years combined. For each iteration, we randomized the observations (return status vs admixture) and calculated the mean for a sample size consistent with our observed group. We calculated the 95% confidence interval of the admixture for the returning fish using the empirical distribution of permuted means by determining the range between the 2.5th and 97.5th percentiles. This interval provides insight into whether the observed means deviated from the values expected under the hypothesis of no difference between the admixture of out-migrating smolts and the survivors of the marine phase.

#### Changes in admixture between smolts and adults

While the number of fish sampled as smolts and returning as adults to the river was low (2–10 per year out of 183–187 smolts sampled per year), which could limit the power to detect a relationship between admixture and marine survival, the high number of adults sampled in the trap (over 12,000 individuals between 2017 and 2023) offers a unique opportunity to gain further insights into this topic. Indeed, using scale reading, the sea age (usually 1–3 years) and therefore the smolt cohort of each individual adult can be determined. For each smolt cohort, it was tested if the admixture rate of migrating smolts was significantly different to that of all adults returning to spawn in the population. Tests for significant differences in distribution of admixture were performed using the non-parametric two-sided Kolmogorov-Smirnov Test^44^. We used Bonferroni correction^45,46^ to correct for multiple testing when performing comparisons by years.

#### Effect of sex on the influence of admixture on marine survival

Traits such as smolt age and age at maturation have been shown to be affected differently by admixture between the sexes^30,47^. Therefore, we investigated if there were differences in selection on admixture during the sea phase between males and females. To do so, we conducted similar analysis as described above for males and females separately.

#### Effect of smolt size or sea winter age on marine survival

Previous studies have shown that marine survival was higher for larger smolts and for one sea winter fish (1SW) compared to multiple sea winter (MSW) fish e.g., refs. ^28,29^. Additionally, Besnier et al.^30^ demonstrated that higher admixture was correlated with larger smolts and early maturation (1SW fish) in males in the Etne river. Other studies have demonstrated that outmigration day^19^ was affected by admixture and have been shown to have a significant effect on survival^48^. To determine if smolt length,weight or outmigration date could influence marine survival in the present study, we examined them in relation to the admixture rate. Using the individuals that were sampled as smolts, we performed a linear regression of length, weight, condition factor and outmigrating day against admixture rate for all the smolts as well as the smolts that survived the marine phase. All years were combined because of the small sample sizes of sampled smolts returning as adults. Because the sample size of sampled smolts returning as adults was small, we also performed a similar linear regression of back-calculated smolt length obtained from scale reading against admixture rate for all adults. Additionally, we resampled the adult dataset 1000 times without replacement, using the same number of individuals found in the smolt dataset (885), to determine if the sample size was affecting the power to observe a significant relationship between smolt size (using back-calculated smolt length) and admixture. We also investigated if there were noticeable differences in admixture distributions between smolts and individuals that returned to spawn in the river as one sea winter fish, versus 2 or 3 sea winter fish. Finally, using the approach described in Besnier et al.^30^, we examined if the probability of early maturation (1SW vs MSW) was correlated with admixture, using a generalized linear mixed effect model with a binomial distribution and a logit link function, with sex and back-calculated smolt length as covariates, and smolt year as a random effect.

#### Statistics and reproducibility

All statistical analyses were done using R (version 4.4.1). Tests for differences of distribution of admixture between smolt cohorts as smolts and as adults were done using a two-sided Kolmogorov-Smirnov Test^44^, using a significance level of 0.05 and a Bonferroni correction^45,46^ to correct for multiple testing when performing the same comparisons across multiple years. We used a generalized linear mixed effect model with a binomial distribution and a logit link function to determine the effect of admixture on early maturation, adding sex and back-calculated smolt length as fixed effects and smolt year as random effect. We explored several models with various interactions, limited to two-way interactions and selected the model with lowest AIC as the best fit.

### Ethics statement

The current permit (Research permit number 30061) was delivered by the Norwegian Food Safety Authority and is valid for all activities at the field station.

### Reporting summary

Further information on research design is available in the Nature Portfolio Reporting Summary linked to this article.

## Supplementary information

Supplementary Information

Description of Additional Supplementary files

Supplementary data 1

Reporting Summary

## Data Availability

The source data behind the figures in the paper are available on the Norwegian Research Information Repository (NVA): 10.83172/3ep9-vq37. Any remaining information can be obtained from the corresponding author upon reasonable request.

## Code availability

All codes supporting the findings and used to create the figures in the paperhave been deposited to the Norwegian Research Information Repository (NVA): 10.83172/3ep9-vq37.
