# Supplementary material for: No significant change in domestication-admixture during the marine phase of an Atlantic salmon population
Source: Commun Biol. 2026 Apr 16;9:1009. doi: 10.1038/s42003-026-10051-z (PMC13396406; doi:10.1038/s42003-026-10051-z)
Supplement: Supplementary file 1 — Supplementary Information [file 42003_2026_10051_MOESM1_ESM.pdf]

Supplementary material

1. **Resampling**
   When randomly resampling the adult dataset with 885 individuals without replacement, we observed a significant relationship between back-calculated smolt length and admixture in only 25% of the cases, with a clear overrepresentation of the tests with pvalue < 0.05 (supplementary figure S4). This proportion increased as we increased the number of samples, suggesting that sample size affected the power to detect a significant relationship between smolt size and admixture in the dataset of individuals sampled as smolts, most likely due to the high variability in smolt size and the small size effect of admixture on smolt size (Besnier, et al. 2022).

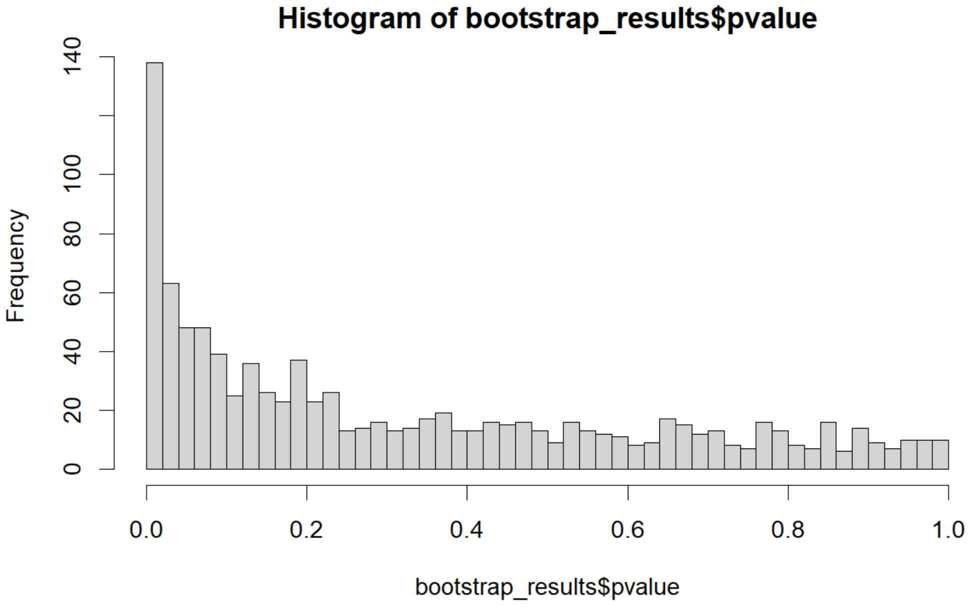

Figure S1: Distribution of p-values after 1000 bootstraps of randomly resampling the adult dataset with 885 individuals without replacement, to determine the relationship between back-calculated smolt length and admixture.

2. **Determination of smolt year**

For individuals whose tag was in the smolt file AND the adult file: error rate for sea age reading from scale 1.78% :

		Smolt year from tagging [Correct year]					
		**2016**	**2017**	**2018**	**2019**	**2020**	**2021**
Smolt Year from scales	**2014**	1					
	**2016**	40	1				
	**2017**	1	60				
	**2018**			43			
	**2019**			1	77		
	**2020**				1	40	
	**2021**						16

Table S1 - Cross-validation of sea age estimated from scales for individuals that were tagged as smolts and returned as adults. Here the number is higher than the sample size for the smolts in the rest of the paper, because most of these individuals were not genotyped and therefore not included in the genetic analysis.

3. Distribution of admixture separated by sex

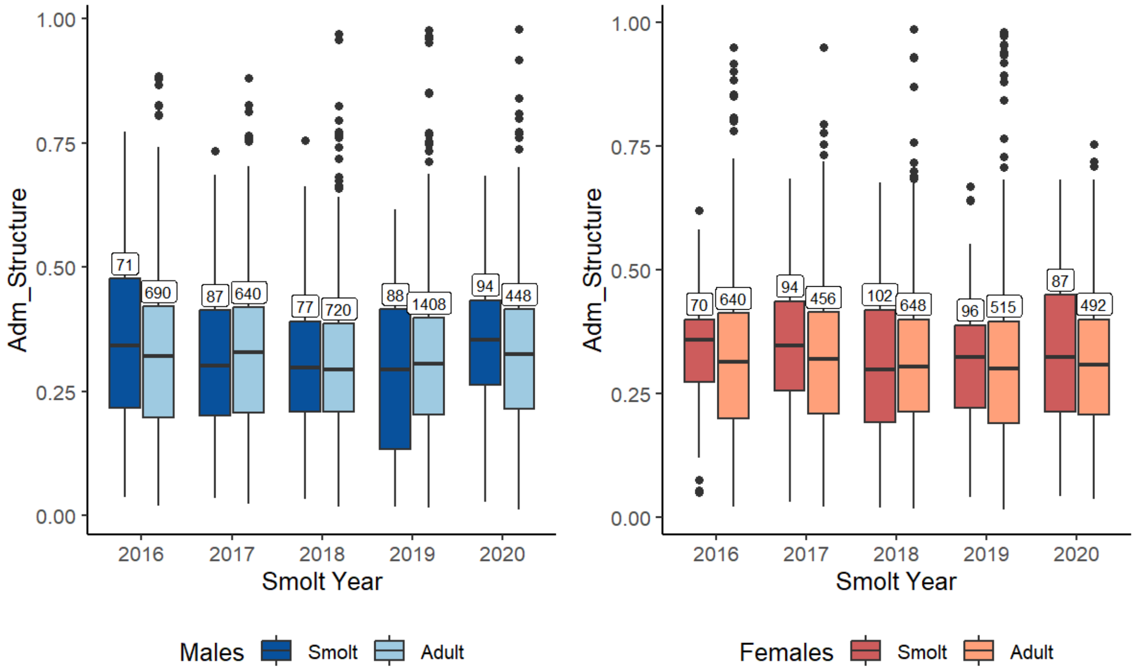

Figure S2 - Comparison of admixture distributions, separated by sex, between smolt and corresponding adult cohorts. Sample sizes are indicated in boxes above each boxplot.

4. Admixture separated by sex and Sea winter age

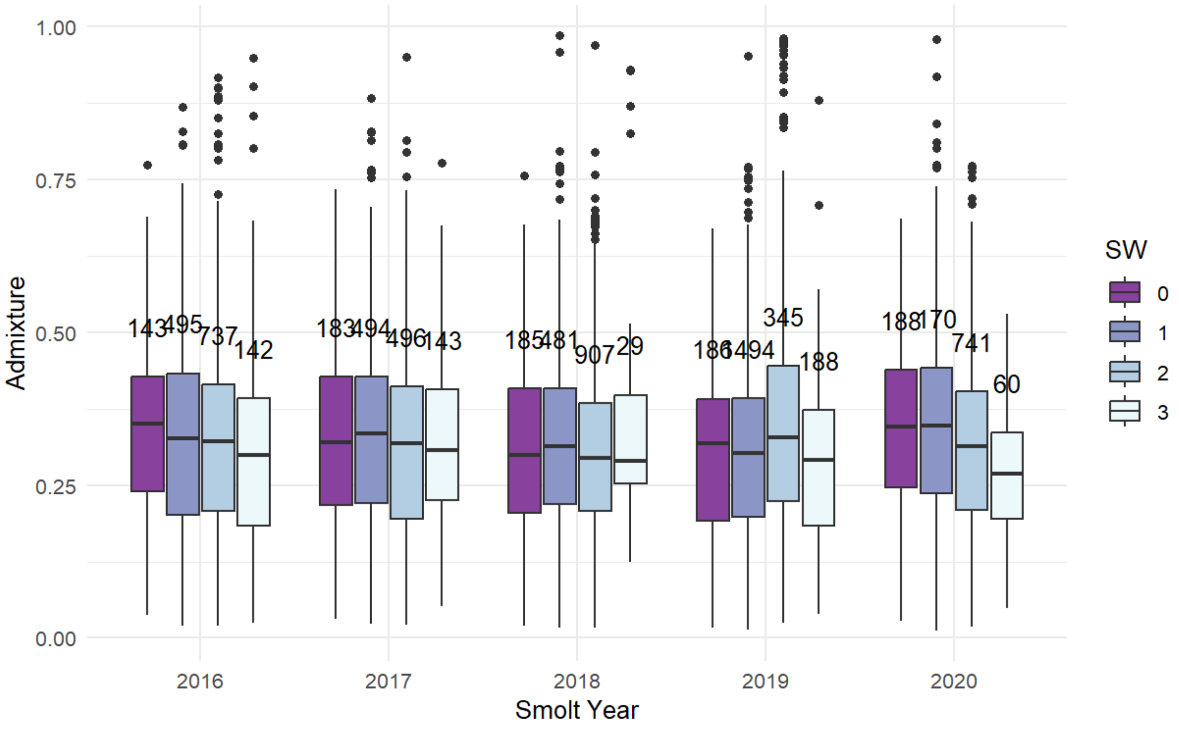

Figure S3a: Distribution of admixture by sea winter (SW) age for all individuals (males and females). SW 0 corresponds to the smolts. Sample sizes are indicated above each boxplot.

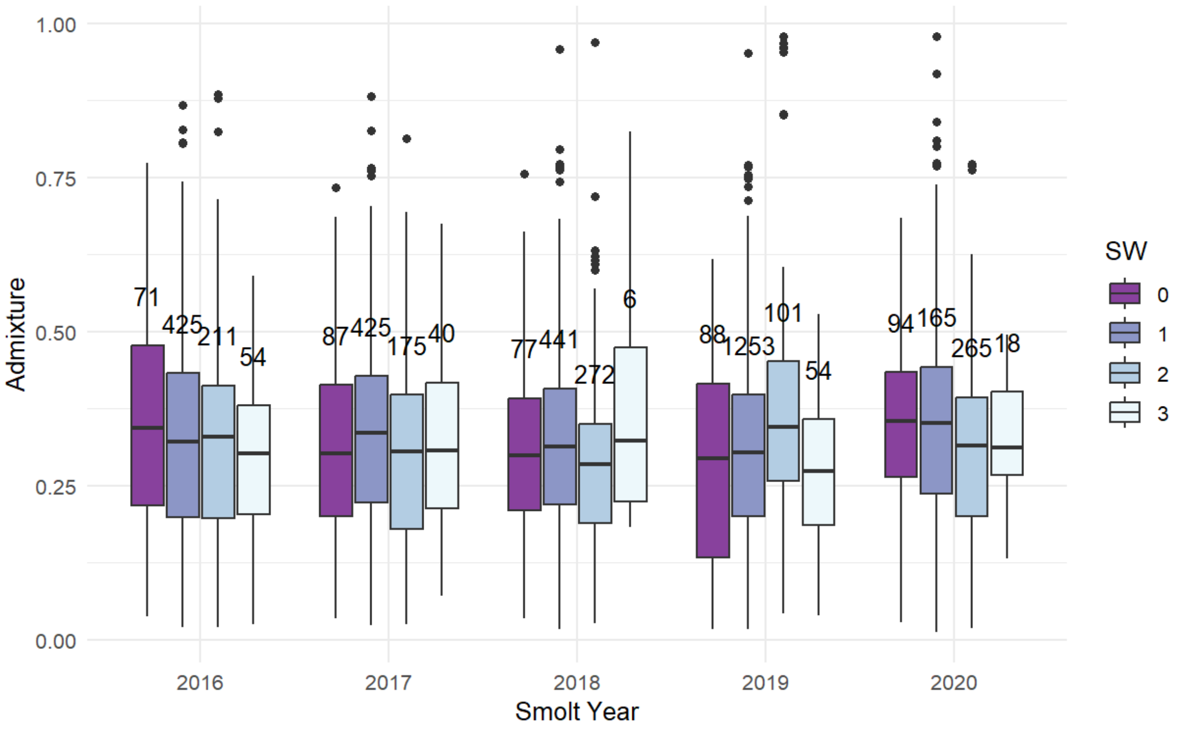

Figure S3b: Distribution of admixture by sea winter (SW) age for the males. SW 0 corresponds to the smolts. Sample sizes are indicated above each boxplot.

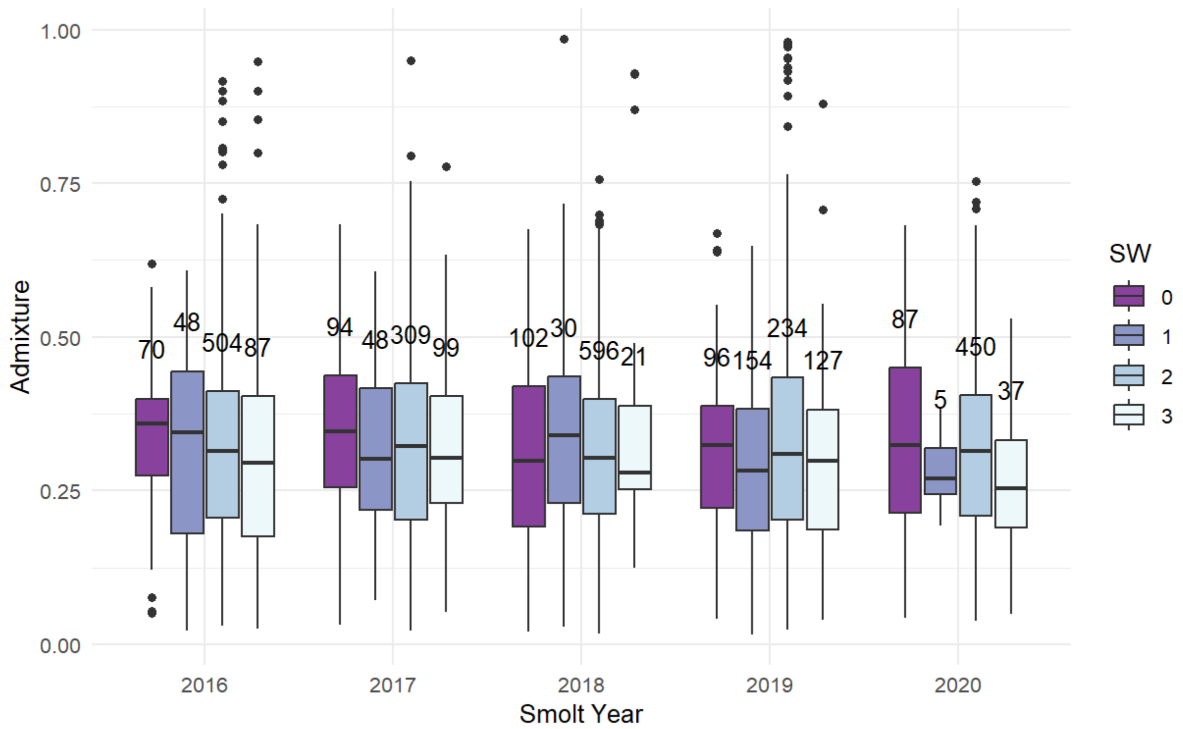

Figure S3c: Distribution of admixture by sea winter (SW) age for the females. SW 0 corresponds to the smolts. Sample sizes are indicated above each boxplot.

1. Effect of admixture on early maturation

Model	ΔAIC
Admixture + SmoltLength + Sex + (1 | SmoltYear)	8.7
Admixture*Sex + SmoltLength + (1 | SmoltYear)	2.2
Admixture*SmoltLength + Sex + (1 | SmoltYear)	7.3
Admixture + SmoltLength*Sex + (1 | SmoltYear)	7.4
Admixture*Sex + Admixture*SmoltLength + (1 | SmoltYear)	1.8
Admixture*Sex + SmoltLength*Sex + (1 | SmoltYear)	0.4
Admixture*SmoltLength + SmoltLength*Sex + (1 | SmoltYear)	6.2
**Admixture*Sex + Admixture*SmoltLength + SmoltLength*Sex + (1 | SmoltYear)**	**0**

TableS2 – Model selection results

```
Fixed effects:
                                  Estimate Std. Error z value Pr(>|z|)    
(Intercept)                       -2.27804    0.41603  -5.476 4.36e-08 ***
Adm_Structure                     -0.51154    0.41766  -1.225  0.22066    
Used_sexM                          2.67137    0.16944  15.765  < 2e-16 ***
Smolt.lengde.scaled               -0.18930    0.09537  -1.985  0.04714 *  
Adm_Structure:Used_sexM            1.38487    0.49016   2.825  0.00472 ** 
Adm_Structure:Smolt.lengde.scaled  0.32095    0.20811   1.542  0.12302    
Used_sexM:Smolt.lengde.scaled     -0.15266    0.07778  -1.963  0.04969 *  
---
Signif. codes:  0 ‘***’ 0.001 ‘**’ 0.01 ‘*’ 0.05 ‘.’ 0.1 ‘ ’ 1
```

Table S3 – Results of best model retained

2. Proportion of smolts sampled

Year	Estimated smolt production*	Individuals PIT-tagged	Proportion of smolts PIT-tagged	Individuals used for genetic analysis	Proportion of smolts used for genetic analysis
2016	40325	1277	0.03167	185	0.00459
2017	42395	2264	0.05340	187	0.00441
2018	72149	2129	0.02951	183	0.00254
2019	67553	2060	0.03049	187	0.00277
2020	86156	2755	0.03198	185	0.00215

Table S4 – Proportion of emigrating smolts sampled for this study. *: Estimates of smolt production in the Etne River from Aune A. (2024) Comparing smolt production estimation methods in Atlantic salmon (*Salmo salar*) [Master's thesis, University of Bergen] Bergen Open Research Archive, https://hdl.handle.net/11250/3164015

3. Comparison SNP chip and SNP panel

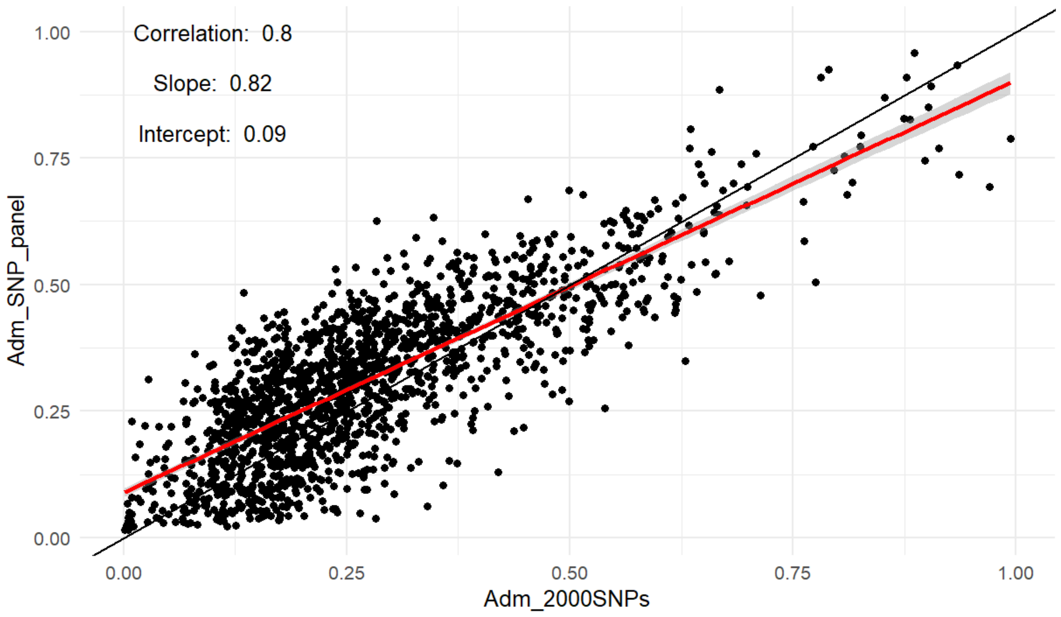

Figure S4 - Estimation of admixture with 2000 SNPs as in Besnier et al. 2022 (x-axis) and with the 130SNP panel in this study (y-axis)

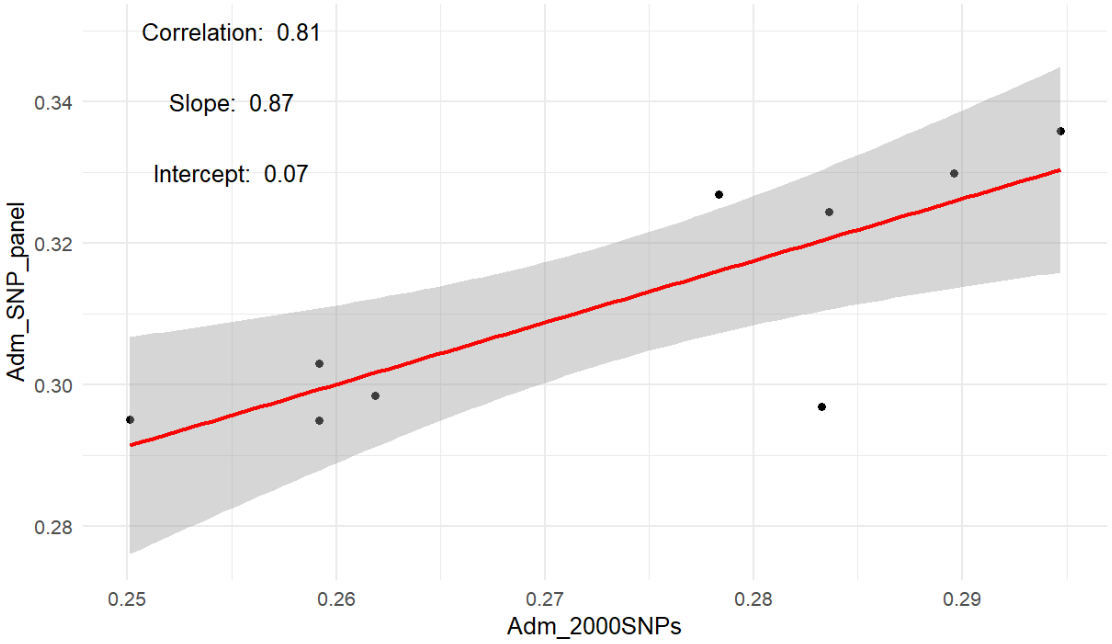

Figure S5 - Comparison of the estimation of mean admixture per year at the population level for all returning adults with 2000 SNPs as in Besnier et al. 2022 (x-axis) and with the 130 SNP panel (y-axis)

In the present study, we used a subset of SNPs from Besnier, et al. (2022) to estimate individual admixture. The observed admixture rate estimated (31%) in Etne is higher than what has been estimated before with a more extensive set of loci from a 50k SNP chip (24%, Besnier, et al. 2022). However, using a reduced panel of loci, as has been done in the present study, has allowed us to greatly increase the sample size, which in turn have enabled us to gain insights into questions we would not be able to address if we were to only rely on more expensive technologies like SNP chips. While we acknowledge the fact that the reduced SNP panel may slightly overestimate the absolute individual admixture rate, we determined that the relative comparisons and changes in admixture rate across years and cohorts, which is the focus of this study, is still valid.

Locus name	F-sequence	R-sequence	Reference	Multiplex	PCR_Program
SSsp2210	AAGTATTCATGCACACACATTCACTGC	CAAGACCCTTTTTCCAATGGGATTC	Paterson et al. (2004)	1	A
SSspG7	CTTGGTCCCGTTCTTACGACAACC	TGCACGCTGCTTGGTCCTTG	Patterson et al. (2004)	1	A
SsaD144	TTGTGAAGGGGCTGACTAAC	TCAATTGTTGGGTGCACATAG	King et.al (2005)	1	A
Ssa202	CTTGGAATATCTAGAATATGGC	GTTCATGTGTTAATGTTGCGTG	O'Reilly et al. (1996)	1	A
Sp2201	TTAGATGGTGGGATACTGGGAGGC	CGGGAGCCCCATAACCCTACTAATAAC	Patersson et al. (2004)	1	A
SsaD157	ATCGAAATGGAACTTTTGAATG	GCTTAGGGCTGAGAGAGGAATAC	King et.al (2005)	1	A
Ssa289	CTTTACAAATAGACAGACT	GTCATACAGTCACTATCATC	McConnell et al. (1995)	2	B
Ssa14	CCTTTTGACAGATTTAGGATTTC	CAAACCAAACATACCTAAAGCC	McConnell et al. (1995)	2	B
Ssa171	TTATTATCCAAAGGGGTCAAAA	GAGGTCGCTGGGGTTTACTAT	O'Reilly et al. (1996)	2	B
Sp2216	GGCCCAGACAGATAAACAAACACGC	GCCAACAGCAGCATCTACACCCAG	Paterson et al. (2004)	2	B
Sp1605	CGTAATGGAAGTCAGTGGACTGG	CTGATTTAGCTTTTTAGTGCCCAATGC	Paterson et al. (2004)	2	B
Sex_Exon2	TGATGGATGGGATCCCCGTCATCTCTCTCCCAAAG	TAGAGCTTAAAACCACTCCACCCTCCATGAGGGA	Eisbrenner et al. (2014)	2 or 3	B
Sex_Exon4	AGTTGGAACGCTTCAGCAGAGCAGATGG	AGATTGGTGCACTGAGTGATGAGTCTTGTCC	Eisbrenner et al. (2014)	2 or 3	B
SSsp3016	GACAGGGCTAAGTCAGGTCA	GATTCTTATATACTCTTATCCCCAT	Genbank no. AY372820	3	B
SsaF43	AGCGGCATAACGTGCTGTGT	GAGTCACTCAAAGTGAGGCC	Sanchez et al. (1996)	3	B
SSa197	TGGCAGGGATTTGACATAAC	GGGTTGAGTAGGGAGGCTTG	O'Reilly et al. (1996)	3	B
SsaD486	TCGCTGTGTATCAGTATTTTGG	ACTCGGATAACACTCACAGGTC	King et al. (2005)	3	B
MHC1	AGGAAGGTGCTGAAGAGGAAC	CAATTACCACAAGCCCGCTC	Grimholt et al. (2002)	3	B
MHC2	GATGGCAAAGAGGAAAGTGAG	TTGTTATGCTCTACCTCTGAA	Stet et al. (2002)	3	B
SsOSL85	TGTGGATTTTTGTATTATGTTA	ATACATTTCCTCCTCATTCAGT	Slettan et al. (1995)	3	B
EST28	CACAGGCACACACTCCTCAT	GTTTCAGGTGAAGAGCATGACCAA	Vasemägi et al. (2005)	4	C
EST19-KA2R	CGCTTCCTGGACAAAAATTA	GTTTCATCTCTGTCATTCTCTTGC	Vasemägi et al. (2005)	4	C
Ssa407	TCGTGACTACTAAGTCTTTGACCA	GTTTGTGTAGGCAGGTGTGGAC	Cairney et al. (2000)	4	C
SSleer15-1	CATGTGCGTGTGCTTTTACAG	GTTTTCTGCATGTAGAACCCTGACC	GenBank U86708	4	C
SLEEN82	CATGGAGAATCCCACTTTCTTA	GTTTCAGGGAGTGATATGGGACATAA	GenBank U86706	4	C
Sleel53	TGATTTGTTGCCTGCTGCTTCC	GTTTCCTGCTGCCCACATCATCC	GenBank U86704	4	C
Ssa412	GTGGAGATACACAGCACTTA	GTTTCTTGGTTAGTACCGGACATG	Cairney et al. (2000)	5	C
Ssa405	CTGAGTGGGAATGGACCAGACA	GTTTACTCGGGAGGCCCAGACTTGAT	Cairney et al. (2000)	5	C
Ssa98	GCAGTCCTTACCTGTGTGATTA	GTTTGGTAGTGATCTGGAGAGTGC	O'Reilly et al. (1996)	5	C
Ssosl25	ATCTACACAGCTCCTGGTGGCAG	GTTTCATGTAATGGGTCGAGAGAAGTG	Slettan et al. (1995)	5	C
SSsp2215	GGTCAGTCAGTCACACCATGC	GTTTAGGTGTCCTGCCGGTCAAT	Paterson et al. (2004)	5	C
EST107	AGCGTTACGTCGAATCCAA	GTTTCTCATGGAGGGTGGAAGTGT	Vasemägi et al. (2005)	5	C
EST68	TGACACTGTGGCCTGTCTCT	GTTTGAGTTCTGGGTTATTTATTCACA	Vasemägi et al. (2005)	5	C

Table S5 – Primers for microsatellites genotyping. PCR programs mentioned are described in Table S6

PCR_Program A

94°C	4min	
94°C	50s	x28
55°C	50s	
72°C	80s	
72°C	10min	
4°C	∞	

PCR_Program B

94°C	4min	
94°C	50s	x26
55°C	50s	
72°C	80s	
72°C	10min	
4°C	∞	

PCR_Program C

95°C	150s	
95°C	25s	x10
58°C	30s	
72°C	25s	
95°C	25s	x29-30
53°C	30s	
72°C	25s	
72°C	600s	
12°C	120s	
4°C	∞	

Table S6 – PCR programs for microsatellite genotyping
