# Supplementary material for: No significant change in domestication-admixture during the marine phase of an Atlantic salmon population
Source: Commun Biol. 2026 Apr 16;9:1009. doi: 10.1038/s42003-026-10051-z (PMC13396406; doi:10.1038/s42003-026-10051-z)
Supplement: Supplementary file 2 — Description of Additional Supplementary files [file 42003_2026_10051_MOESM2_ESM.pdf]

Description of Additional Supplementary files

File name: Supplementary Data 1

Description: Primers for the SNPs genotyped in this study
