# Supplementary material for: No significant change in domestication-admixture during the marine phase of an Atlantic salmon population
Source: Commun Biol. 2026 Apr 16;9:1009. doi: 10.1038/s42003-026-10051-z (PMC13396406; doi:10.1038/s42003-026-10051-z)
Supplement: Supplementary file 4 — Reporting Summary [file 42003_2026_10051_MOESM4_ESM.pdf]

nature portfolio

Corresponding author(s): Marine S.O. Brieuc

Last updated by author(s): Mar 2, 2026

Reporting Summary

Nature Portfolio wishes to improve the reproducibility of the work that we publish. This form provides structure for consistency and transparency in reporting. For further information on Nature Portfolio policies, see our Editorial Policies and the Editorial Policy Checklist.

Statistics

For all statistical analyses, confirm that the following items are present in the figure legend, table legend, main text, or Methods section.

n/a	Confirmed	
☐	☒	The exact sample size (n) for each experimental group/condition, given as a discrete number and unit of measurement
☐	☒	A statement on whether measurements were taken from distinct samples or whether the same sample was measured repeatedly
☐	☒	The statistical test(s) used AND whether they are one- or two-sided<br>*Only common tests should be described solely by name; describe more complex techniques in the Methods section.*
☐	☒	A description of all covariates tested
☐	☒	A description of any assumptions or corrections, such as tests of normality and adjustment for multiple comparisons
☐	☒	A full description of the statistical parameters including central tendency (e.g. means) or other basic estimates (e.g. regression coefficient) AND variation (e.g. standard deviation) or associated estimates of uncertainty (e.g. confidence intervals)
☒	☐	For null hypothesis testing, the test statistic (e.g. F, t, r) with confidence intervals, effect sizes, degrees of freedom and P value noted<br>*Give P values as exact values whenever suitable.*
☒	☐	For Bayesian analysis, information on the choice of priors and Markov chain Monte Carlo settings
☒	☐	For hierarchical and complex designs, identification of the appropriate level for tests and full reporting of outcomes
☒	☐	Estimates of effect sizes (e.g. Cohen's d, Pearson's r), indicating how they were calculated

Our web collection on statistics for biologists contains articles on many of the points above.

Software and code

Policy information about availability of computer code

Data collection	No software was used
Data analysis	R version 4.4.1<br>Structure 2.3.4<br>COLONY v.2.0.7.1

For manuscripts utilizing custom algorithms or software that are central to the research but not yet described in published literature, software must be made available to editors and reviewers. We strongly encourage code deposition in a community repository (e.g. GitHub). See the Nature Portfolio guidelines for submitting code & software for further information.

Data

Policy information about availability of data

All manuscripts must include a data availability statement. This statement should provide the following information, where applicable:

- Accession codes, unique identifiers, or web links for publicly available datasets
- A description of any restrictions on data availability
- For clinical datasets or third party data, please ensure that the statement adheres to our policy

All data supporting the findings of this study will available within the paper and its Supplementary Information will be deposited in the publically available Brage IMR repository.

Research involving human participants, their data, or biological material

Policy information about studies with human participants or human data. See also policy information about sex, gender (identity/presentation), and sexual orientation and race, ethnicity and racism.

Reporting on sex and gender	Research did not invole human participants, their data, or biological material
Reporting on race, ethnicity, or other socially relevant groupings	Research did not invole human participants, their data, or biological material
Population characteristics	Research did not invole human participants, their data, or biological material
Recruitment	Research did not invole human participants, their data, or biological material
Ethics oversight	Research did not invole human participants, their data, or biological material

Note that full information on the approval of the study protocol must also be provided in the manuscript.

Field-specific reporting

Please select the one below that is the best fit for your research. If you are not sure, read the appropriate sections before making your selection.

☐ Life sciences ☐ Behavioural & social sciences ☒ Ecological, evolutionary & environmental sciences

For a reference copy of the document with all sections, see nature.com/documents/nr-reporting-summary-flat.pdf

Ecological, evolutionary & environmental sciences study design

All studies must disclose on these points even when the disclosure is negative.

Study description	Here we aimed to characterize the effect of admixture on marine survival in a highly domestication-admixed wild salmon population
Research sample	All samples used in this study are Atlantic salmon individuals collected in the Etne River in Norway. Some individuals were collected as smolts as they left the river while adults were collected as they came back to the river to spawn.
Sampling strategy	For the adults, all adults have to go through a fish trap on their way to the river to be sampled before being released upstream from the trap to continue their migration. All adults were therefore sampled.<br>Only a portion of the smolts were PITtagged (800/year), but only around 185 per year were genotyped and used in this study. The numbers of smolts tagged were chosen based on the known low marine survival to ensure that we get back enough fish to ensure statistical power.
Data collection	Data was collected by multiple agents over many years. Smolts were anesthetised with Benzocaine (0.5 mL to 2 Lwater) before sampling and PITtagging. Adults are not anesthetised as sampling lasts for less than 2 mins and is done directly in the river, so we do not need to disturb the fish any more than needed with anesthetic.
Timing and spatial scale	Timing for adults: entire migration season at the permanently installed trapping facility.<br>Timing for smolts: The trap is set out during the smolt migration, which we know is between april and June, and needs to be out before the fishing season starts (now, 15 June).
Data exclusions	Individuals with more than 50% missing values when genotyped with the 31 microsatellite loci or with the 130 SNP panel were excluded from the analysis.
Reproducibility	Not relevant in the study as we are examining natural observations - there was no experiments conducted
Randomization	Not relevant in the study as we are examining natural observations - there was no experiments conducted
Blinding	Not relevant in the study as we are examining natural observations - there was no experiments conducted

Did the study involve field work? ☒ Yes ☐ No

Field work, collection and transport

Field conditions	Not relevant because sampling occurred over many years in many different weather conditions

Location	Fjord and River laboratory field station in Etne, Norway (59°40'12.7"N 5°56'44.4"E)
Access & import/export	The trap has been installed since 2013. The current permit (Research permit number 30061) was delivered by the Norwegian Food Safety Authority and is valid for all activities at the field station.
Disturbance	*Describe any disturbance caused by the study and how it was minimized.*

Reporting for specific materials, systems and methods

We require information from authors about some types of materials, experimental systems and methods used in many studies. Here, indicate whether each material, system or method listed is relevant to your study. If you are not sure if a list item applies to your research, read the appropriate section before selecting a response.

Materials & experimental systems

n/a	Involved in the study
☒	☐ Antibodies
☒	☐ Eukaryotic cell lines
☒	☐ Palaeontology and archaeology
☐	☒ Animals and other organisms
☒	☐ Clinical data
☒	☐ Dual use research of concern
☒	☐ Plants

Methods

n/a	Involved in the study
☒	☐ ChIP-seq
☒	☐ Flow cytometry
☒	☐ MRI-based neuroimaging

Animals and other research organisms

Policy information about studies involving animals; ARRIVE guidelines recommended for reporting animal research, and Sex and Gender in Research

Laboratory animals	The study did not involve laboratory animals
Wild animals	All animals used in this study were Atlantic salmon. They were either smolts (outmigrating) or adults (migrating back to the river to spawn). They were all caught in the fish traps and anesthetized before sampling and being PITtagged (smolts only). They were then released from the trap on the site of collection to continue their migrations.
Reporting on sex	Sex of all individuals was done my genotyping the sex marker for Atlantic salmon.<br>Information about sex was used to determine if there were any differences in survival between males and females
Field-collected samples	The study did not involve laboratory work from live field-collected samples
Ethics oversight	Research permit number 30061 was delivered by the Norwegian Food Safety Authority and is valid for all activities at the field station

Note that full information on the approval of the study protocol must also be provided in the manuscript.

Plants

Seed stocks	No plants were used in this study
Novel plant genotypes	No plants were used in this study
Authentication	No plants were used in this study
